# Early Detection of Cognitive, Language, and Motor Delays for Low-Income Preterm Infants: A Brazilian Cohort Longitudinal Study on Infant Neurodevelopment and Maternal Practice

**DOI:** 10.3389/fpsyg.2021.753551

**Published:** 2021-10-28

**Authors:** Nadia C. Valentini, Luana S. de Borba, Carolina Panceri, Beth A. Smith, Renato S. Procianoy, Rita C. Silveira

**Affiliations:** ^1^Escola de Educação Física, Fisioterapia e Dança, Universidade Federal do Rio Grande do Sul, Porto Alegre, Brazil; ^2^Escola de Fisioterapia, Centro Universitário Ritter dos Reis (UniRitter), Porto Alegre, Brazil; ^3^Hospital de Clínicas de Porto Alegre, Porto Alegre, Brazil; ^4^Developmental Neuroscience and Neurogenetics Program, The Saban Research Institute, Division of Research on Children, Youth, and Families, Children’s Hospital Los Angeles, Department of Pediatrics, Keck School of Medicine, University of Southern California, Los Angeles, CA, United States; ^5^Escola de Medicina, Universidade Federal do Rio Grande do Sul, Porto Alegre, Brazil

**Keywords:** premature birth, gestational age, risk factors, child development, cognitive development, language development, motor development

## Abstract

**Aim:** This study examined the neurodevelopment trajectories, the prevalence of delays, and the risks and protective factors (adverse outcomes, environment, and maternal factors) associated with cognitive, motor, and language development for preterm infants from 4– to 24-months.

**Method:** We assessed 186 preterm infants (24.7% extremely preterm; 54.8% very preterm; 20.4% moderate/late preterm) from 4– to 24-months using the Bayley Scales of Infant Development – III. Maternal practices and knowledge were assessed using the Daily Activities of Infant Scale and the Knowledge of Infant Development Inventory. Birth risks and adverse outcomes were obtained from infant medical profiles.

**Results:** A high prevalence of delays was found; red flags for delays at 24-months were detected at 4– and 8-months of age. The neurodevelopmental trajectories showed steady scores across time for cognitive composite scores for extremely- and very-preterm infants and for language composite scores for the extremely- and moderate/late-preterm; a similar trend was observed for the motor trajectories of moderate/late preterm. Changes over time were restricted to motor composite scores for extremely- and very-preterm infants and for cognitive composite scores for moderate/late preterm; declines, stabilization, and improvements were observed longitudinally. Positive, strong, and significant correlations were for the neurodevelopment scores at the first year of life and later neurodevelopment at 18 and 24 months. The cognitive, language, and motor composite scores of extremely and very preterm groups were associated with more risk factors (adverse outcomes, environment, and maternal factors). However, for moderate/late preterm infants, only APGAR and maternal practices significantly explained the variance in neurodevelopment.

**Discussion:** Although adverse outcomes were strongly associated with infant neurodevelopment, the environment and the parents’ engagement in play and breastfeeding were protective factors for most preterm infants. Intervention strategies for preterm infants should start at 4– to 8-months of age to prevent unwanted outcomes later in life.

## Introduction

Annually worldwide, about 30 million infants are born premature (World Health Organization [WHO], 2018). In Brazil, 11.5% of all births are premature (16% extremely; 10% very; 74% moderate/late). Prematurity is the leading cause of childhood death in the first 5 years of life (Leal et al., 2016; França et al., 2017). It is also highly associated with neurodevelopmental impairments (Goldenberg et al., 2008; Nishimura et al., 2016). Extremely preterm infants have high rates of severe neurological impairments (17–59%), such as intellectual disability (5–36%) and cerebral palsy (9–18%) (Jarjour, 2015). Low scores for language, motor (You et al., 2019), and cognitive (Hodel et al., 2017) skills are reported for moderate/late preterm infants.

Several risk factors negatively affect preterm infants. Adverse outcomes, such as cerebral injuries (Linsell et al., 2015; Månsson et al., 2015), leukomalacia (Lin et al., 2020), and periventricular hemorrhage (PIVH) (Soares et al., 2017)are frequently reported for preterm infants. Although, more prevalent in the lower gestational age groups (Soares et al., 2017; Lin et al., 2020). Often those infants need mechanical ventilation (Månsson et al., 2015) and long-term hospitalization (Soares et al., 2017). Besides the adverse outcomes, environmental factors are related to infant neurodevelopment (Son and Morrison, 2010; Patra et al., 2016; Pereira et al., 2016; Asztalos et al., 2017; Borba et al., 2017; Hou et al., 2020; Lin et al., 2020). Low socioeconomic status (Son and Morrison, 2010; Linsell et al., 2015; Pereira et al., 2016; Borba et al., 2017; Yaari et al., 2018; Lin et al., 2020) and maternal formal education (Patra et al., 2016) have been negatively associated with preterm cognitive, motor, and language development. Moreover, appropriate maternal practices and interactions, safe and roomy physical space, opportunities to explore the home, and experiences of active play with parents during the first few years of life promote child development (Son and Morrison, 2010; Pereira et al., 2016; Borba et al., 2017; Panceri et al., 2017; Rocha et al., 2019; Rodrigues et al., 2019; Valentini et al., 2020). Family and home environment are vital contributors to the child’s physical well-being, impacting the child throughout life (Son and Morrison, 2010), and may even more vital for a child exposed to the adverse outcomes of prematurity.

Although prenatal care services have advanced in recent years, the early diagnosis of preterm infants who have adverse outcomes (Bhutta et al., 2014) and the establishment of risk and protective factors (Yaari et al., 2018; Lin et al., 2020) is still a challenge in several countries (Jahan et al., 2021). LMIC (low-moderate income countries) have used more accessible and faster tests such as Denver II (Santos et al., 2008); an assessment with low sensitivity for children younger than 8 months old. Furthermore, the longitudinal effects of the risk factors associated with the adverse outcomes for different preterm groups (extremely, very, and moderate late preterm), the stabilization and changes in cognitive, language, and motor trajectories, and the prevalence of delays across those groups still lack investigation. Besides, subtle impairments are underdiagnosed, so as the intervention referral (Hodel et al., 2017).

This study aimed to examine, longitudinally, the neurodevelopment trajectories (changes and stabilization of composite scores), the prevalence of delays, and the risks and protective factors (adverse, environmental, and maternal factors) associated with cognitive, motor, and language development for extremely, very, and moderate/late preterm infants from 4– to 24-months of corrected age (CA). We expected that the extreme and the very preterm infants would have a significantly lower neurodevelopment rate, higher prevalence of delays, and more risk factors associated with those outcomes. Moderate/late preterm infants will also develop unsteadily over time; however, they would have less prevalence of delays and fewer risk factors associated with the outcomes. Also, we expect that protective factors would be associated with better developmental scores in all groups.

## Materials and Methods

### Participants

Preterm infants (*N* = 242) from a cohort longitudinal study in a public hospital in Brazil were initially enrolled in the present study. The assessments were carried out from July 2016 to December 2019. The hospital ethical review board approved the research, and parents signed informed consent. Data from the preterm infants who attended at least two longitudinal assessments were analyzed (*N* = 186; 24.7% extremely preterm: gestational age – GA < 28 weeks; 54.8% very preterm: GA 28-to-32-weeks; 20.4% moderate/late preterm: GA 32-to-37-weeks). All infants had several adverse outcomes and environmental risk factors that imply a high risk of disability. The extremely and very preterm groups stayed for long time in NICU (Neonatal Intensive Care Unit), needed mechanical ventilation (60.8% extremely; 29.5% very), parenteral nutrition (>90%), and blood transfusion (extremely > 80%; very > 40%). Extremely (43.5%) and very (23.6%) preterm infants had combined early sepsis, later sepsis, and PIVH. Most infants were from low-income families (monthly income: 63.8% less than 200 dollars; 28.3% 400 dollars).

### Instruments and Procedures

**FIGURE 1:**
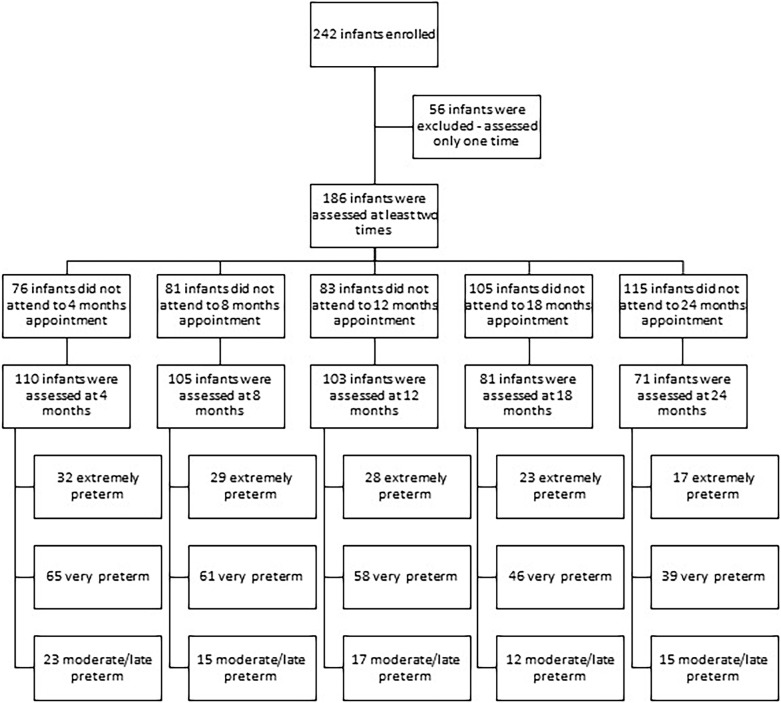
Flow chart of infants enrolled in the study.

Birth factors and adverse outcomes were obtained from medical records. The Bayley Scales of Infant and Toddler Development-III (BSID-III) (Bayley, 2006), adopting CA, was used to assessing cognitive, language, and motor development. Composite score and performance categorization, a score that take in consideration the child age (very superior ≥ 130; superior: 120–129; high average: 110–119; average: 90–109; low average: 80–89; borderline: 70–79; extremely low ≤ 69) were reported; composite scores ≥ 90 were accepted as in the typical range, and composite score ≤ 89 were considered as delay (Bayley, 2006). The Daily Activities of Infant Scale – DAIS (Bartlett et al., 2008) was used to assess parental practices. The Knowledge of Infant Development Inventory – KIDI (Nobre-Lima et al., 2014) was used to assess the parent’s knowledge about child development. Developmental assessments were conducted at 4–, 8–, 12–, 18–, and 24-months of CA, in the presence of parents or legal guardians. Some children did not attend some appointments and therefore did not have data at all time points. Figure 1 presents a flow chart for infants enrolled in the study and the infants that discontinue the participation by groups and assessment period. Two trained professionals conducted the assessments independently, with a high inter-rater agreement (ICC > 85).

### Data Analysis

The sample size calculation was performed based on the results of the composite scores of the Bayley-III scales throughout the follow-up, using the WinPEPI program (Programs for Epidemiologists for Windows), version 11.43. Considering a significance level of 5%, power of 80%, and a minimum effect size of 0.6 standard deviations between the assessments, a minimum total of 24 children per group was obtained (Motta and Wagner, 2006).

Regarding risk and protective factors, several measures were used to compare groups. *T*-test and ANOVA with Tukey *post hoc* were used to compare groups’ means, and in the case of asymmetries, the Kruskal–Wallis and Dunn in the case of asymmetric distribution were used as a *post hoc*. The Chi2 was used to compare groups for categorical variables with the residual test as a *post hoc*. Pearson or Spearman correlations were used to verify associations between quantitative variables. For the Pearson and Spearman correlations recognizes cut off were used (strong > 0.60; moderate: 0.30–0.60; weak: <0.30).

The GEE (General Equation Estimation) model with the Least Significant Difference adjustment was conducted to compare BSID-III scores for intra- and inter-groups longitudinally. The linear model (symmetric distribution), the gamma model (asymmetric distribution), or the logistic model (categorical, ordinal variables) were adopted. The logistic model in the GEE was used to compare the frequencies of delays. The GEE was used to examine the longitudinal data since it involves repeated measurements of cognitive, language, and motor scores that tend to correlate with one another, which must be taken into proper account. The GEE models allow for substantial flexibility in specifying the correlation structure within cases and offer the potential for valuable substantive insights into the nature of that correlation (Burton et al., 1998; Zorn, 2001). The GEE also allowed for the intra-group comparison in each assessment period, preventing the bias of multiple independent comparisons. Pearson correlations were used to verify the association within each domain and age longitudinally.

Backward multivariate linear regression was used to examine the risks and protective factors (exposure variables) associated with infants’ outcomes (cognitive, motor, language) by groups (extremely preterm *N* = 46; very preterm *N* = 101; and moderate/late preterm *N* = 38). The scores used in the multivariate regression analysis were the ones obtained in the last assessment of each infant; 47% (*n* = 71) of all infants had their last assessment at 24 months, 30% (*n* = 45) of them at 18 months, and 23% (*n* = 36) of them at 12 months; the extraction method was used in the regression model to control for confounding factors. The criterion for entering the variable in the multivariate model was a *p*-value < 0.20 in the bivariate analysis. The criterion for maintaining the variable in the final model was a *p*-value < 0.10 in the multivariate analysis, following recognized guidelines for Backward regression (Hair et al., 1998). Cohen *f*^2^ was used as a measure of effect size for the regression (cut off: small ≤ 0.15; moderate 0.15 to 0.34; large > 0.35).

## Results

### Preliminary Analysis

In the present study we assume that the data is missing at random, as some children have assessments at two, three, four, or five time points randomly. Nevertheless, group comparisons were conducted with all variables between the two groups (children with all assessments and children with one, two or three missing assessment). Similarity was observed between groups for birth factors and adverse outcomes (GA *p* = 0.503; birth weight *p* = 0.058; seizures *p* = 0.422; PIVH *p* = 0.271; leukomalacia *p* = 0.245; days of invasive mechanical ventilation *p* = 0.055; NICU stay *p* = 0.493; parenteral nutrition *p* = 0.335), socioeconomic factors (maternal formal education *p* = 0.664; paternal formal education *p* = 0.256; mother age *p* = 0.648; father age *p* = 0.357; length of breast-feeding *p* = 0.159; socioeconomic status *p* = 0.365) and Bayley scores at all ages, 4 months (cognitive *p* = 0.207; language *p* = 0.454; motor *p* = 0.328), 8 months (cognitive *p* = 0.981; language *p* = 0.771; motor *p* = 0.622), 12 months (cognitive *p* = 0.303; language *p* = 0.927; motor *p* = 0.964), 18 month (cognitive *p* = 0.250; language *p* = 0.810; motor *p* = 0.253) and at 24 months (cognitive *p* = 0.957; language *p* = 0.785; motor *p* = 0.549). These results support the GEE robustness even with missing data.

Table 1 presents the sample demographics (n and %) by groups.

**TABLE 1:** Sample demographic by groups.

	Preterm groups *N*(%)		
Clinical outcomes	Extremely preterm	Very preterm	Moderate/late
	
	GA < 28 weeks	GA 28-to-32 weeks	GA 32-to-37 weeks
	
	*N* = 46	*N* = 101	*N* = 38
NICU stay 3–4 weeks	–	7 (6.9)	16 (42.1)
5–6 weeks	–	29 (28.4)	15 (39.5)
7–8 weeks	1 (2.2)	30 (29.4)	3 (7.9)
9–10 weeks	7 (15.2)	13 (12.7)	3 (7.9)
11–12 weeks	6 (13.0)	7 (6.9)	1 (2.6)
13–14 weeks	7 (15.2)	6 (5.9)	–
15–16 weeks	13 (28.3)	3 (2.9)	–
17 > weeks	12 (26.0)	7 (6.9)	–
NICU Seizures Yes	18 (39.1)	16 (15.7)	6 (15.8)
Mechanical ventilation No	18 (39.2)	72 (70.5)	31 (81.6)
1–2 weeks	12 (26.1)	25 (24.5)	7 (18.4)
3–4 weeks	7 (15.2)	2 (2.0)	–
5–6 weeks	3 (6.5)	–	–
7 > weeks	6 (13.0)	3 (3.0)	–
Oxygen therapy No	18 (39.2)	56 (54.9)	22 (58)
1–2 weeks	13 (28.3)	34 (33.2)	13 (34.2)
3–4 weeks	6 (13.0)	5 (4.9)	1 (2.6)
5–6 weeks	2 (4.3)	3 (3.0)	1 (2.6)
7 > weeks	7 (15.2)	4 (4.0)	1 (2.6)
Parenteral nutrition No	4 (8.7)	8 (7.8)	12 (31.6)
1–2 weeks	12 (26.1)	61 (59.8)	24 (63.2)
3–4 weeks	13 (28.2)	24 (23.5)	1 (2.6)
5–6 weeks	11 (23.9)	5 (4.9)	–
7 > weeks	6 (13.1)	4 (4.0)	1 (2.6)
Leukomalacia Yes	4 (8.7)	4 (3.9)	3 (7.9)
Periventricular No	26 (56.5)	78 (76.4)	28 (73.7)
Hemorrhage I	8 (17.5)	17 (16.7)	10 (26.3)
II	7 (15.2)	4 (3.9)	0
III	3 (6.5)	2 (2.0)	0
IV	2 (4.3)	1 (1.0)	0
Early sepsis Yes	39 (84.8)	60 (58.8)	16 (42.2)
Late sepsis Yes	37 (80.4)	50 (49.0)	7 (18.4)
Transfusion Yes	41 (89.1)	44 (43.2)	6 (15.8)
N^0^ blood transfusion 1–3	19 (41.3)	36 (35.3)	7 (18.4)
4–6	12 (26.1)	6 (5.9)	–
7–9	7 (15.2)	1 (1.0)	–
10–12	3 (6.5)	1 (1.0)	–
Pre-eclampsia Yes	15 (32.6)	31 (30.4)	18 (47.4)

### Group Comparisons: Birth Risks, Adverse Outcomes, and Environmental Factors

Table 2 presents the participants’ birth risks and adverse outcomes by groups and statistical results. Significant differences were found across preterm groups. Overall, the extremely preterm group had more negative birth risks and adverse outcomes than the other two groups, followed by the very preterm group.

**TABLE 2:** Birth risks and clinical outcomes and group comparisons.

Birth factors and clinical outcomes			Preterm groups			
			Extremely	Very	Moderate/late	*p*
Prenatal care visits		MD (P25 – P75)	4 (3–4)^a^	5 (4–7)^b^	7 (4–8)^b^	0.001
Preeclampsia		*N* (%) – Yes	15 (33.3)	31 (32)	18 (58.1)*	0.027
Twin pregnancy		*N* (%) – Yes	9 (20.0)	29 (28.7)	5 (13.9)	0.160
Birth delivery	Cesarean	*N* (%)	34 (75.6)	67 (69.1)	25 (73.5)	0.700
	Vaginal		11 (24.4)	30 (30.9)	9 (26.5)	
Sex	Boys	*N* (%)	23 (50)	59 (57.8)	18 (47.4)	0.456
	Girls	*N* (%	23 (50)	43 (42.2)	20 (52.6)	
Gestational age (weeks)		*M* (*SD*)	26.5 (1.1)^a^	30.1 (1.3)^b^	33.4 (1.4)^c^	<0.001
Length at birth (cm)		*M* (SD)	33.7 (3.2)^a^	38.9 (3.2)^b^	41.0 (3.6)^c^	<0.001
Cephalic perimeter at birth (cm)		*M* (SD)	24 (2.0)^a^	27.4 (2.2)^b^	29.5 (1.8)^c^	<0.001
Weight at birth (grams)		*M* (SD)	838 (195)^a^	1333 (328)^b^	1717 (622)^c^	<0.001
Weight (grams)	500–1000 g	*N* (%)	36 (80)*	13 (13.3)	–	<0.001
	1000–1500 g		9 (20)	67 (68.4)*	21 (67.7)	
	1500–2000 g		–	18 (18.4)	10 (32.3)*	
Apgar 5th min		MD (P25 – P75)	7 (6–8)^a^	8 (8–9)^b^	8 (7–9)^ab^	0.001
NICU stay (days)		MD (P25 – P75)	99 (77–116)^c^	49 (36–69)^b^	30 (26–38)^a^	<0.001
Invasive mechanical ventilation (days)		MD (P25 – P75)	6 (0–26)^b^	0 (0–2)^a^	0 (0–0)^a^	< 0.001
Oxygen therapy (days)		MD (P25 – P75)	5 (0–25)^b^	0 (0–7)^a^	0.5 (0–5)^a^	0.014
Periventricular hemorrhage		*N* (%) – Yes	20 (45.5)	24 (25.3)	10 (37.0)	0.053
Leukomalacia		N (%) – Yes	4 (9.3)	4 (4.2)	3 (11.1)	0.323
NICU Seizures		N (%) – Yes	18 (39.1)*	16 (16.3)	6 (18.8)	0.008

*MD, median; M, mean; SD, standard deviation.*

*^a,b,c^Different letters: significant differences at Tukey (symmetric distribution) or Dunn (asymmetric distribution), p < 0.050 (quantitative variables).*

*Statistically significant association by the residual test adjusted to 5% significance, *p* < 0.050 (categorical variables).

Table 3 presents the environmental factors by groups and the statistical results. The only significant differences across groups were related to paternal ages; the fathers of moderate/late preterm infants were significantly older than the fathers of the other two groups.

**TABLE 3:** Environment factors and group comparisons.

Environmental factors		Preterm groups			
		Extremely	Very	Moderate/late	*p*
Maternal age (years)	*M* (*SD*)	26.3 (6.6)	27.9 (6.6)	29.6 (7.1)	0.093
Paternal age (years)	*M* (*SD*)	30.9 (8.3)^ab^	30.0 (8.2)^a^	35.4 (9.9)^b^	0.015
Maternal formal education	*N* (%)				
Incomplete middle		6 (14.3)	12 (13.8)	8 (27.6)	0.154
Middle complete		10 (23.8)	12 (13.8)	5 (17.2)	
Incomplete high school		6 (14.3)	10 (11.5)	1 (3.4)	
High school degree		13 (31.0)	41 (47.1)	9 (31)	
Incomplete undergraduate		6 (14.3)	5 (5.7)	2 (6.9)	
Undergraduate degree		1 (2.4)	7 (8.0)	4 (13.8)	
Paternal formal education	*N* (%)				
Incomplete Middle		7 (18.9)	16 (18.6)	6 (23.1)	0.802
Middle complete		9 (24.3)	17 (19.8)	4 (15.4)	
Incomplete high school		6 (16.2)	15 (17.4)	6 (23.1)	
High school degree		13 (35.1)	30 (34.9)	8 (30.8)	
Incomplete undergraduate		1 (2.7)	3 (3.5)	0 (0.0)	
Undergraduate degree		0 (0.0)	5 (5.8)	2 (7.7)	
Graduate degree		1 (2.7)	0 (0.0)	0 (0.0)	
Parents lived together	N (%)	24 (72.7)	50 (89.3)	14 (82.4)	0.132
Family income (R$)	MD (P25–P75)	1600 (1200–2000)	2000 (1200–3000)	1900 (1175–3775)	0.526
N^0^ children at home	MD (P25–P75)	1 (0–2)	1 (0–2)	1 (0–2)	0.630
N^0^ adolescent at home	MD (P25–P75)	0 (0–0)	0 (0–0)	0 (0–0)	0.859
Breast feeding (months)	MD (P25 – P75)	6 (0.3–12.3)	3 (0–7)	4 (1–12)	0.341

*M, mean; MD, median; SD, standard deviation.*

*^*a,b*^Different letters: significant differences at Tukey (symmetric distribution), *p* < 0.050.*

### Cognitive, Language, and Motor Trajectories and Prevalence of Delay by Groups

**FIGURE 2:**
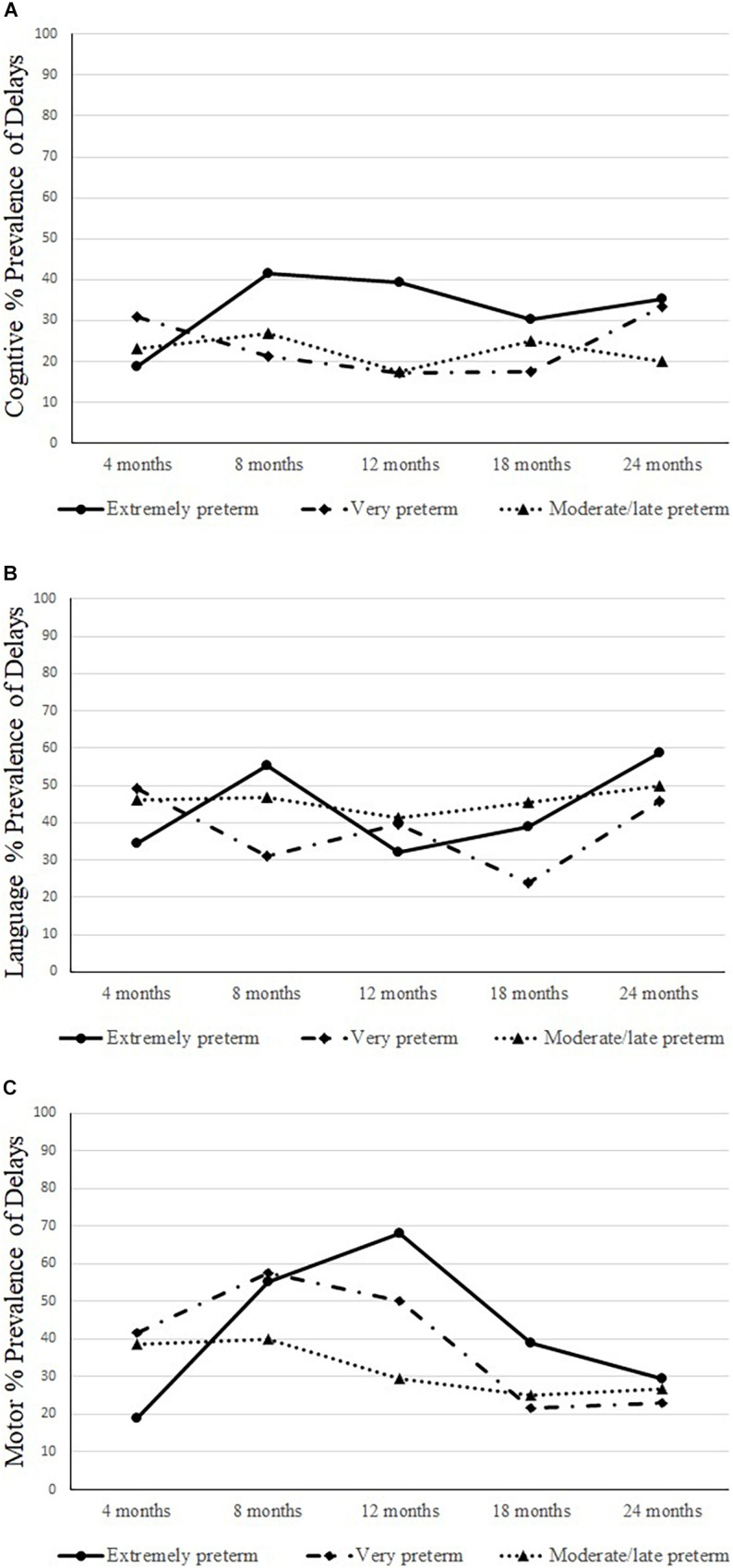
Prevalence of cognitive **(A)**, language **(B)**, and motor **(C)** delays longitudinally by groups.

Figure 2 presents the prevalence for cognitive (1a), language (1b), and motor (1c) delays by groups longitudinally.

Table 4 presents the correlation within each neurodevelopment domain longitudinally by groups.

**TABLE 4:** Neurodevelopment correlations within each neurodevelopment domain in each age.

Groups	Cognitive correlations				Language correlations				Motor correlations			
	4 m	8 m	12 m	18 m	4 m	8 m	12 m	18 m	4 m	8 m	12 m	18 m
**Correlations coefficients longitudinally in each neurodevelopment domain for composite scores**												
**Extremely preterm**												
4 m	–	–	–	–	–	–	–	–	–	–	–	–
8 m	0.24	–	–	–	0.17	–	–	–	0.45*	–	–	–
12 m	0.29	0.70**	–	–	0.03	0.18	–	–	0.56**	0.65**	–	–
18 m	0.59*	0.55*	0.80**	–	0.21	0.67**	0.50	–	0.60*	0.63**	0.92***	–
24 m	0.47	0.11	0.71**	0.77*	0.46	0.48	0.71**	0.24	0.57*	0.51	0.81**	0.82**
**Very preterm**												
4 m	–	–	–	–	–	–	–	–	–	–	–	–
8 m	0.37	–	–	–	0.37*	–	–	–	0.26	–	–	–
12 m	0.70***	0.63***	–	–	0.43**	0.27	–	–	0.56***	0.58***	–	–
18 m	0.52***	0.44*	0.81***	–	0.24	0.37*	0.60***	–	0.32	0.62***	0.75***	–
24 m	0.22	0.45*	0.67***	0.62**	0.23	0.54**	0.61**	0.68***	0.04	0.67***	0.67***	0.83***
**Moderate/late preterm**												
4 m	–	–	–	–	–	–	–	–	–	–	–	–
8 m	0.30	–	–	–	0.62	–	–	–	0.01	–	–	–
12 m	0.53	0.49	–	–	0.45	0.27	–	–	0.10	0.45	–	–
18 m	0.12	0.76*	0.80*	–	0.15	0.67	0.13	–	0.15	0.69	0.93**	–
24 m	0.26	0.30	0.33	0.75	0.54	0.28	0.89*	0.97***	0.91*	0.60	0.89*	0.62

Pearson correlations, *p < 0.050, **p < 0.01, ***p < 0.001; m, months.

#### Extremely Preterm

The neurodevelopmental trajectories results showed that the cognitive composite scores at 4–, 12–, 18–, and 24-months scores were slightly above 90; however, at 8-month-old, the scores were lower; non-significant changes were observed longitudinally. The language composite scores at 4– to 18-months were around 90; at 24-months, the scores were lower; non-significant differences were found longitudinally. The motor development composite scores at 4– and 24-months were above 90, and at 8–, 12–, and at the18-months scores were significantly lower than at 4– and 24-months. Significant, positive, and strong correlations were found within each neurodevelopment domain and age. The correlations showed that the cognitive composite scores observed at 8 months were more robust than those at 12 months, and the same trend was observed for the 12 months with 18 and 24 months. A similar trend was observed for language scores – stronger associations were found for 8 and 18 months and between 12 months with 24 months. For motor scores, strong correlations were observed even early, at 4 months and 18 and 24 months, also between 8 months and 12 and 24 months, and between 12 months and 24 months. Therefore, for this group, neurodevelopment scores observed in the first year of life were strongly related to the 18- and 24-months scores. The prevalence of delays found for cognitive development at 8–, 12–, 24-months was ± 40%, for language development at 8- and 24-months was ± 60%; and for motor development at 8- and 12-months was ± 50%.

#### Very Preterm

The neurodevelopmental trajectories results showed that the composite scores for cognitive were above 94 across all ages; no significant changes were found longitudinally. Regarding language, the composite scores at 8–, 12–, 18–, and 24-months were around 90; at 4-months of age, the score was lower; the composite score at 18 months was significantly higher than at 4-months. The motor scores at 4–, 12–, 18–, and 24-months were above 90, and at 8-months were lower; significantly higher composite scores were found at 18- and 24-months compared to 8-months. Significant, positive, and strong correlations were found within each neurodevelopment domain and age. The correlations showed that the cognitive composite scores observed at 4 months were strongly related to the scores at 12 months, and the same trend was observed between the scores at 8 months with 12 months and between 12 months and 18 and 24 months. For language, the stronger associations were found between 12 months and 18 and 24 months. For motor scores, stronger correlations were observed between 8 months and 18 and 24 months, between 12 months and 18 and 24 months, and between 18 and 24 months. Therefore, for this group, neurodevelopment scores observed in the first year of life were strongly related to the scores at 18 and 24 months scores. The prevalence of delays found for cognitive development at 4 and 24 months was ± 30%; for language development at 4– and 24-months was ± 50%; and for motor development at 8- and 12-months was ± 50%.

#### Moderate/Late Preterm

The neurodevelopmental trajectories results showed that the composite cognitive scores at 4– and 18-months were above 90, however, significantly lower than in other ages. The language composite scores at 4–, 12–, 18–, and 24-months were above 90, and at 8-months, the scores were lower; non-significant differences were found longitudinally. The motor scores at 4–, 12–, 18–, and 24-months were above 90, and at 8-months, the score was lower; non-significant differences were found. Significant, positive, and strong correlations were found within each neurodevelopment domain and age. The correlations showed that the cognitive composite scores observed at 8 months were strongly related to the scores at 12 months, and the same trend was observed between 12 months and 18 months. For language, the stronger associations were found between 12 and 24 months and between 18 and 24 months. For motor scores, stronger correlations were observed between 4 and 24 months and between 12 months and 18 and 24 months. Therefore, for this group, neurodevelopment scores observed in the first year of life were strongly related to the scores at 18 and 24 months. The prevalence of delays found for cognitive development at 8- and 18-months was ± 30%; for language at 4–, 8–, and 24-months was ± 50%; and for motor development at 4– and 8-months was ± 40%.

### Groups by Time Comparisons: Motor Trajectories and Prevalence of Delays

#### Motor Trajectories

The GEE analysis showed non-significant interactions for groups by time for cognitive (*p* = 0.307) and language (*p* = 0.106) composite scores. A significant group-by-time interaction was found for motor composite scores (*p* = 0.004). Higher scores were found for extremely preterm compared to the very preterm at 4-months (*p* = 0.013), and at 12-months (*p* = 0.007), the extremely preterm showed lower composite scores compared to moderate/late preterm. Table 5 presents the motor trajectories for cognitive, language, and motor scores by groups and the GEE results.

**TABLE 5:** Developmental trajectories: cognitive, language, and motor composite scores by groups and results.

Preterm groups	*M*(*SD*) Longitudinal (months) Bayley scores and statistical results					
	4 m	8 m	12 m	18 m	24 m	*p*

*N* total	110	105	103	81	71	
**Extremely**	**(*n* = 32)**	**(*n* = 29)**	**(*n* = 28)**	**(*n* = 23)**	**(*n* = 17)**	
Cognitive	96.3 (16.3)	89.1 (14.0)	92.5 (17.9)	91.7 (16.6)	93.2 (17.6)	0.533
Language	91.9 (9.6)	91.9 (14.3)	90.9 (14.5)	90.4 (12.0)	88.2 (16.4)	0.743
Motor	97.5 (13.6)^c^	87.7 (16.2)^ab^	81.7 (21.9)^a^	87.9 (22.8)^ab^	92.0 (19.0)^bc^	<0.001
**Very**	**(*n* = 65)**	**(*n* = 61)**	**(*n* = 58)**	**(*n* = 46)**	**(*n* = 39)**	
Cognitive	94.2 (18.3)	96.6 (14.1)	99.2 (17.8)	97.5 (14.3)	95.3 (17.9)	0.220
Language	89.3 (11.9)^a^	93.2 (14.2)^ab^	92.9 (15.8)^ab^	96.7 (14.7)^b^	91.8 (17.1)^ab^	0.020
Motor	92.0 (18.5)^ab^	87.5 (16.8)^a^	92.5 (20.5)^ab^	97.3 (20.5)^b^	96.1 (17.4)^b^	0.006
**Moderate/Late**	**(*n* = 13)**	**(*n* = 15)**	**(*n* = 17)**	**(*n* = 12)**	**(*n* = 15)**	
Cognitive	92.7 (14.1)^a^	99.7 (14.3)^ab^	101.8 (16.2)^b^	91.3 (20.5)^a^	96.7 (20.8)^ab^	0.047
Language	93.4 (8.1)	89.8 (15.5)	95.2 (20.1)	90.3 (16.4)	92.4 (15.3)	0.866
Motor	95.2 (13.4)	88.7 (19.0)	94.7 (19.6)	95.4 (20.9)	97.2 (18.6)	0.641

*M, mean; SD, standard deviation; Bayley average scores ≥ 90.0.*

*^a,b,c^GEE least significant difference: statistical significance is represented in the table using the letters “a,” “b,” and “c”: notice that if the letters differ between the two time points within a group (a and b/c), it is an indication that there is a statistically significant difference between the time points; however, if the letters remain the same between the time points (a, a, a or b, b, b or c, c), no statistically significant results were found for those time points; groups by time differences are described in the results section significance p < 0.050.*

#### Prevalence of Delays

*Regarding the prevalence of cognitive* delays by groups, non-significant group-by-time interaction was found (*p* = 0.307); group effect was found at 12-months with a higher prevalence of delays for the extremely preterm group than in the very preterm (*p* = 0.035). Regarding language, a non-significant group by time interaction was found (*p* = 0.106); group effect was significant at 8-months with a higher prevalence of delays in the extremely preterm group than in the very preterm (*p* = 0.029). Regarding motor delays prevalence, a significant group by time interaction was found (*p* = 0.004). A higher prevalence of motor delays was found for the very preterm infants compared to the extremely preterm at 4-months (*p* = 0.013); however, at 12-months a higher prevalence of motor delays was found in the extremely preterm group than in the moderate/late preterm infants (*p* = 0.007). Increases in the prevalence of motor delays in the extremely preterm infants from 4– to 12-months (*p* < 0.001) were also found.

### Cognitive, Language, and Motor Predictors by Groups

Correlations results for birth factors, adverse outcomes, environmental risks, maternal practices, and maternal knowledge with cognitive, language, and motor scores by groups are presented in Table 6. Significant correlations, for quantitative variables, were found for birth risks (weight, length, Apgar 5th minute), adverse outcomes (NICU stays, mechanical ventilation, oxygen therapy), environmental factors (paternal age, formal maternal education, family income), and maternal practices (breastfeeding, postures during changing, and active play). Nominal variables, such as PIVH and NICU seizures, due to its nature (dichotomous variables) were analyzed using differences between means for cognitive, language, and motor composite scores for each option (yes or no). The extremely preterm group, infants with PIVH had significant lower composite scores for motor (*M* = 78.7; *SD* = 22.1; *p* = 0.002) and cognitive (*M* = 84.8; *SD* = 16.1; *p* = 0.002) development compared to infants without PIVH. Infants with NICU seizures had significant lower composite scores for motor (*M* = 77.7; *SD* = 20.4; *p* = 0.001), cognitive (*M* = 81.9; *SD* = 15.0; *p* = 0.000), and language (*M* = 84.7; *SD* = 13.6; *p* = 0.021) development compared to infants without NICU seizures. For the very preterm group, infants with PIVH had similar neurodevelopment scores to those without PIVH, and infants with NICU seizures had significant lower composite scores for motor (*M* = 80.7; *SD* = 22.9; *p* = 0.017), cognitive (*M* = 85.0; *SD* = 17.4; *p* = 0.004), and language (*M* = 81.9; *SD* = 15.2; *p* = 0.003) development compared to those without NICU seizures. Moderate/late preterm group, infants with PIVH or NICU seizures had similar neurodevelopment scores to those without PIVH or NICU seizures.

**TABLE 6:** Associations: birth, clinical outcomes, maternal practices, and knowledge.

Birth, clinical outcomes and maternal practices, and knowledge		Correlations coefficients								
		Extremely preterm			Very preterm			Moderate/Late preterm		
		Cognitive	Motor	Language	Cognitive	Motor	Language	Cognitive	Motor	Language

*N* total			46			101			38	
**Birth and clinical outcomes**										
Birth weight (g)	*r*	0.19	0.22	0.09	0.24*	0.21*	0.02	–0.10	–0.10	–0.02
Birth length (cm)	*r*	0.46**	0.36**	0.17	0.25*	0.23*	0.09	–0.07	–0.06	0.06
Cephalic perimeter (cm)	*r*	0.26	0.15	0.08	0.16	0.11	–0.06	–0.08	0.02	0.01
Apgar 5th min	*r*_s_	0.22	0.23	–0.06	0.17	0.07	–0.08	0.34*	0.33	0.39*
NICU stay (days)	*r*_s_	−0.60***	−0.51***	–0.04	−0.41***	−0.46***	−0.29**	–0.15	0.04	−0.34*
IVM days	*r*_s_	−0.30*	−0.50***	–0.12	−0.24*	−0.28**	–0.14	–0.02	0.18	–0.09
Oxygen therapy (days)	*r*	–0.03	–0.12	0.15	–0.20	−0.28**	−0.26*	–0.22	–0.03	–0.33
Maternal age – years (*r*)	*r*	0.10	0.03	–0.12	0.02	0.11	0.14	–0.14	–0.08	–0.22
Paternal age – years (*r*)	*r*	0.11	0.03	–0.10	0.02	0.06	0.02	–0.17	–0.19	−0.37*
Maternal formal education	*r*_s_	0.22	0.05	0.35*	0.18	0.14	0.27*	0.25	0.02	0.04
Paternal formal education	*r*_s_	0.05	0.04	0.12	0.14	0.15	0.11	0.10	–0.02	0.17
Family income (Reals)	*r*_s_	0.18	0.38*	0.11	0.09	0.20	0.32**	0.24	0.19	0.11
N^0^ Prenatal care visits	*r*_s_	–0.24	0.03	–0.22	0.01	0.08	0.10	–0.18	–0.17	–0.26
N^0^ children at home	*r*_s_	0.26	0.29	–0.18	–0.14	–0.12	–0.11	0.07	0.15	0.06
N^0^ adolescents at home	*r*_s_	–0.14	–0.28	–0.18	–0.12	0.00	–0.16	0.01	–0.12	–0.26
**Maternal practice**										
Breastfeeding (months)	*r*_s_	0.49**	0.16	0.04	0.32*	0.37**	0.33*	0.50*	0.17	0.42
**Maternal practice - daily activities of infant scale from 4 to 12 months**										
Feeding	*r*	0.18	0.08	–0.09	–0.04	–0.08	–0.09	0.25	–0.11	0.02
Bathing	*r*	0.14	–0.02	–0.19	0.18	0.03	0.02	0.35	–0.04	0.09
Changing cloths	*r*	0.09	–0.21	–0.15	0.23*	0.11	–0.01	0.26	–0.11	0.05
Carry in the lap	*r*	0.15	0.12	–0.13	0.17	0.02	0.01	0.26	–0.06	0.02
Calm/quite play	*r*	0.22	0.09	–0.05	0.18	–0.02	–0.00	0.35	–0.04	0.09
Active play	*r*	0.30	0.15	–0.12	0.28*	0.13	0.04	0.37	0.01	0.16
Outside stroll/walks	*r*	0.26	–0.00	–0.07	0.19	0.03	–0.03	0.28	0.00	0.02
Sleeping	*r*	0.17	0.18	–0.14	0.04	–0.14	–0.09	–0.08	–0.03	0.06
**Maternal knowledge - Knowledge of Infant Development Inventory from 4 to 12 months**										
Total score	*r*	0.29	0.29	0.17	–0.04	–0.09	–0.04	–0.25	–0.04	–0.27

*p < 0.05, **p < 0.01, ***p < 0.001.

*r, Pearson correlation; *r*_s_, Spearman correlations; IMV, Invasive Mechanical Ventilation. Dichotomous variables are presented in means and standard deviation. Significant correlations were analyzed using a *T*-test.*

All assumptions for the multivariate regression model were met. Appropriate values of skewness and kurtosis (Sk ≤ 2.0 and Ku ≤ 3.8), the residuals of the regression were normality distributed, and the results of variance inflation factor clear suggested no multicollinearity in the data (values between 1.01 and 1.12). Therefore, the backward multivariate regression was conducted. The regression analysis showed different predictors for each group. For the extremely preterm group, 43.9% of the variance in cognitive development was explained by the PIVH and NICU seizures, 20.6% of the variance in language by the formal maternal education and NICU seizures, and 78.8% of the variance in the motor by active play, NICU seizures, Apgar, formal maternal education, and the number of children at home. All variables were significant predictors of neurodevelopment (Beta from 0.27 to 0.54), and all with moderate and large effect size.

For the very preterm group, 36.3% of the variance in cognitive development was explained by NICU stay, breastfeeding, maternal education, 27.9% of the variance in language by the number of prenatal care visits, maternal education, and NICU seizures, 47.1% of the variance in the motor by active play, parents living together, oxygen therapy, and maternal education. Some variables fail to remain significant in the model, but several predictors were found (Beta values from 0.28 to 0.47).

For the moderate/late premature group, fewer variables explained the model variance; 21.3% of the cognitive development was explained by the Apgar 5th minute, and 28.3% and 32.3% of the language and motor development variance, respectively, were explained by breastfeeding. All factors significantly predict neurodevelopment (Betas values from 0.49 to 0.57). Table 7 presents the results from linear multivariate regression by groups.

**TABLE 7:** Linear multivariate regression by groups.

Predictors by groups	b (CI 95%)	Beta	*p*	*R* ^2^	ES (*f*^2^) *R*^2^	Adjusted *R*^2^	ES (f^2^) adjusted *R*^2^
**Extremely preterm (*N* = 46)**							
**Cognitive**							
Periventricular hemorrhage	–13.7(–26.4 to –1.1)	–0.38	0.035	43.9%	0.78^###^	38.6%	0.63^###^
NICU seizures	–16.7(–29.1 to –4.3)	–0.47	0.011				
**Language**							
Maternal formal education	3.5 (0.40 to 6.5)	0.34	0.025	20.6%	0.26^##^	16.4%	0.20^##^
NICU seizures	–8.5 (–17.0 to 0.00)	–0.29	0.050				
**Motor**							
DAIS Active play	12.7 (6.8 to 18.5)	0.54	<0.001	78.8%	3.72^###^	72.20%	2.60^###^
NICU seizures	–19.8 (–30.1 to –9.4)	–0.49	0.001				
Apgar 5th minute	3.8 (0.30 to 7.4)	0.27	0.037				
Maternal formal education	5.1 (1.4 to 8.7)	0.35	0.010				
N^0^ children at home	6.4 (0.30 to 12.5)	0.27	0.041				
**Very preterm (*N* = 101)**							
**Cognitive**							
NICU stay	–0.20 (–0.40 to –0.10)	–0.42	<0.004	36.3%	0.57^###^	31.20%	0.45^###^
Breastfeeding (months)	0.90 (0.10 to 1.7)	0.30	0.033				
Maternal formal education	2.7 (0.40 to 5.8)	0.24	0.082				
**Language**							
N^0^ Prenatal care visits	1.8 (0.20 to 3.5)	0.28	0.028	27.9%	0.39^###^	23.30%	0.30^##^
Maternal formal education	4.2 (1.5 to 6.9)	0.39	0.003				
NICU seizures	–11.5 (–25.1 to 2.1)	–0.21	0.096				
**Motor**							
DAIS Active play	10.6 (5.0 to 16.1)	0.47	<0.001	47.1%	0.89^###^	41.30%	0.70^###^
Parents living together	19.3 (4.8 to 33.7)	0.33	0.010				
Oxygen therapy (days)	–0.30 (–0.60 to 0.01)	–0.25	0.057				
Maternal formal education	2.8 (–0.20 to 5.9)	0.24	0.064				
**Moderate/late preterm (*N* = 38)**							
**Cognitive**							
Apgar 5th minute	7.2 (1.0 to 13.3)	0.49	0.024	21.3%	0.27^##^	14.70%	0.17^##^
**Language**							
Breastfeeding (months)	1.0 (0.10 to 1.9)	0.53	0.028	28.3%	0.39^###^	23.50%	0.31^##^
**Motor**							
Breastfeeding (months)	1.2 (0.10 to 2.3)	0.57	0.043	32.3%	0.48^###^	26.20%	0.35^###^

*DAIS, Daily Activities of Infant Scale; CI, Confident Interval; *p* < 0.05; ES, Effect Size of Cohen (*f*^2^) was used as a measure of effect size for the regression (cut off: ^##^moderate 0.15 to 0.34; ^###^large > 0.35) using effect size calculator for regression: https://www.danielsoper.com/statcalc/calculator.aspx?id=5.*

## Discussion

### Groups Adverse Outcomes, Environment Factors, and Prevalence of Developmental Delays

#### Extremely Preterm

Infant neurodevelopment at 8-months had a similar pattern that was observed again at 24-months. Like our results, a previous study showed that a considerable rate of extremely preterm infants scored below 70 in cognitive, language, and motor development at 2-year-old (Serenius et al., 2013; Månsson et al., 2015); and another study reported comparable results showing that 29–40% of extremely preterm infants had cognitive delays at 2-year-old (Aarnoudse-Moens et al., 2017). The long-term effect may be expected for the extremely preterm infants in the present study since cognitive delay has been reported to reach 40% for infants with similar risk factors at school age (Linsell et al., 2015) and even at adolescence and adulthood (Rogers and Hintz, 2016). In the present study, the prevalence of delays at 24-months was already observed at 8-months.

#### Very Preterm

A similar pattern of delays was found for the overall development at 4– and 24-months of age. Lower prevalence of delays has been reported for very preterm infants at 18-months (16.9% cognitive, 20.6% motor) (Pascal et al., 2018) and at 22-months compared to our study, although in a combined group of extremely and very preterm infants (22% motor, 15% cognitive, 35% language) (Synnes et al., 2017). For infants born very premature, cognitive and language development delays are considered the most prevalent sequels (Linsell et al., 2015; Synnes et al., 2017; Pascal et al., 2018), similar to our study.

#### Moderate/Late Preterm

A higher prevalence of delays for the cognitive, language, and motor outcomes was found at 8-months, compared to the other ages. Less is known about moderate/late preterm regarding development, limiting our capacity to contrast results. However, cognitive impairments have been reported before the age of 3-years (You et al., 2019), and minor discrepancies in the development of executive function at pre-school age (Hodel et al., 2017), similar, in part, with our results, although with older children.

For the three preterm group, a closer look at development trajectories (measured by the composite scores), the prevalence of overall delays (cognitive, language, and motor combined), and the correlations within each domain and age suggested that infant performance at the first year of life, at 8-months, had a similar pattern that was observed again at 24-months, strong associations were found between age and infant performance.

#### Comparisons Between Groups

Overall, extremely preterm infants had a higher prevalence of cognitive and motor (12-months) and language (8-months) delays, and for this group, the prevalence of delays significantly increased from 4– to 12-months. The prevalence of developmental delay is inversely proportional to gestational age and birth weight (Synnes et al., 2017; Pascal et al., 2018), and it was confirmed in the present study. Besides, like previous studies (Linsell et al., 2015; Synnes et al., 2017; Lawlor et al., 2018; You et al., 2019), the prevalence of delays was observed in the first year of life. However, we provided evidence that at the age of 24-months, the delay observed in the early months (4– and 8-months) was recurrent. Therefore, providing intervention programs at the first signs of delay may prevent further compounding impairments in life. Delayed diagnosis and consequently the lack of access to interventions program is a reality in LMIC (Valentini et al., 2020); urgent actions are needed to identify and refer these children even before the manifestation of delays (Valentini et al., 2020; Jahan et al., 2021).

It is essential to notice that, although prematurity is an independent risk factor for developmental adversities, the population of preterm infants shows considerable variability in the severity of their impairments (Rogers and Hintz, 2016), and it was observed in the present study across groups and longitudinally. Variability in premature infants may be due to the factors involved in this process, whether related to the individual’s biology or the environmental context. In the present study, no significant differences were found, between groups, regarding environmental characteristics, such as parents’ age and education, family income, breastfeeding time, and parents’ stable union. Therefore, an explanation for such variability across groups (higher prevalence of motor development delay in very preterm infants) and time (more oscillations in performance in extremely and very preterm groups, first and second, respectively) is probably due to the adverse outcomes for those infants. As observed in the present study (refer to Table 2), more severe complications were found for the extremely preterm and then in the very preterm group, explaining the overall lower scores and delays in neurodevelopment.

### Adverse Outcomes and Infant Neurodevelopment

Different adverse outcomes were associated and were accounted for the variability in neurodevelopment. For extremely, very, and moderate/late preterm groups, the predictors were PIVH, NICU seizures and stay, and Apgar 5th minute.

Periventricular hemorrhage was a strong predictor for cognitive development in the extremely preterm group, similar to previous studies (Mukerji et al., 2015; Soares et al., 2017; Kenyhercz et al., 2020). Moderate to severe cognitive delays are reported for preterm infants diagnosed with mild to severe PIVH; levels III and IV (Mukerji et al., 2015). It is also associated with sepsis worsening the neurological prognosis (Maglione et al., 2018).

During hospitalization, seizures were also a predictor of cognitive, language, and motor development for extremely preterm infants. Neonatal seizures have been reported as significantly related to low motor scores; 23.1% of children who suffered a seizure had a delay in acquiring motor skills (Zavadenko et al., 2018), similar to our results. Here we provided evidence for the association with cognitive and language development. Seizures are the leading cause of brain injuries that affect neurodevelopment. Preterm infants have a fragile nervous system and are not prepared for the extrauterine environment, consequently more susceptible to seizures (de Kieviet et al., 2012).

Prolonged hospitalization, a consequence of several adverse outcomes, was inverse and significantly associated with very preterm cognitive development. Previous studies showed a similar trend; the longer the hospitalization, the more unfavorable outcomes in the cognitive development were found for preterm infants (Veleda et al., 2011; Månsson et al., 2015). Hospital environments restrict infants’ movement, and social interactions (Panceri et al., 2017), excessive expose preterm infants to light, manipulation, interruption of sleep, and painful procedures (Carvalho and Pereira, 2017); all factors combined may have a negative and lasting impact on several areas of infant development (Karasik et al., 2011; Lobo and Galloway, 2013).

Apgar score in the 5th minute of life was predictive of motor development in the extremely preterm group and cognitive development in the moderate/late preterm group. Previous association for mental and gross motor development (de Kieviet et al., 2012) has been reported, in alignment with our results. On the other hand, no associations between Apgar and motor development have been reported for a similar age group (Gampel and Nomura, 2014). Apgar score assesses the newborn’s adverse condition and identifies the need for immediate assistance, the association found in the present study may be explained by severe but punctual factors at birth which may have repercussions on later development.

### Environment Factors and Infant Neurodevelopment

The environmental factors, maternal education, number of children at home, number of prenatal care visits, and parents living together were associated and accounted for the variability in the neurodevelopment of extremely and very preterm groups.

Maternal formal education was a significant predictor of language development for the extremely and very preterm groups, like previous studies (Patra et al., 2016; Asztalos et al., 2017; Yaari et al., 2018; Hou et al., 2020), and motor development for the extremely preterm group, aligned with previous studies (de Kieviet et al., 2012; Yaari et al., 2018). However, in some studies (Patra et al., 2016; Asztalos et al., 2017), these results were also found in late preterm infants; here, we found the relevance of this factor to extremely and very preterm infants.

The parents’ stable union and a shared life in the same house was a predictor for motor development of the very preterm group, a plausible explanation for this finding is a higher motor stimulation quality within environments where both parents live together (Maria-Mengel and Martins Linhares, 2007; Defilipo et al., 2012). Being cared for by both parents is a protective factor for infants living in socioeconomic vulnerability (Maria-Mengel and Martins Linhares, 2007), similar to our sample. Related to this finding, the presence of other children in the domestic environment was a positive predictor of motor development for the extremely preterm group; optimized environmental opportunities for stimulation are observed when more adults or children are around the new infant (Defilipo et al., 2012).

The number of prenatal care visits was a strong predictor for language development in the very preterm group. Previous studies have reported that the lack of prenatal care was associated with deficits in motor development (Brito et al., 2011), physical growth (da Rocha Neves et al., 2016), neonatal mortality and morbidity (Mbuagbaw et al., 2015), and development disorders (Crestani et al., 2013). Less is known regarding language; here, we advanced in the current knowledge, showing the importance of this care for later language development. Prenatal care visits reduce this risk of complications during the pregnancy and provide information about infant care and development, factors that combined could be related to language development.

It is essential to notice that the environmental risk factors could change and affect neurodevelopmental outcomes differently depending on the child’s age. The present study measured its effect on neurodevelopment across the second year of life, from 12 to 24 months, with no distinction of a specific period. Therefore, the results can only be interpreted for this age span.

The scores used in the multivariate regression analysis were the ones obtained in the last assessment of each infant; 47% (*n* = 71) of all infants had their last assessment at 24 months, 30% (*n* = 45) of them at 18 months, and 23% (*n* = 36) of them at 12 months; the extraction method was used in the regression model to control for confounding factors.

### Maternal Practices and Infant Neurodevelopment

Maternal practices were associated with and accounted for the variability in the neurodevelopment of extremely, very, and moderate/late preterm groups. Specifically, opportunities for movement at home and engagement in active play, provided by mothers, were the strongest predictor of motor development for extremely and very preterm groups. Breastfeeding was the stronger predictor for language and motor development for the moderate/late preterm group. Parental appropriate practices that provided infants with toys to play with and physical space to actively move and explore the environment benefit motor (da Rocha Neves et al., 2016; Borba et al., 2017), and language (da Rocha Neves et al., 2016; Hadders-Algra, 2016) development. Active play is characterized by activities with less supportive postures, allowing greater freedom of movement exploration, which may allow for more social interaction and improve language skills. The parent’s perceptions may also explain the relevance of active play toward the preterm infants’ vulnerability and the need to be engaged and facilitate in daily care postures and opportunities that stimulate development (Bartlett et al., 2011; Correa et al., 2019; Rodrigues et al., 2019).

It is essential to notice that maternal practices regarding the quality of the stimuli offered to the infant (e.g., age-appropriate activities, opportunity to explore different postures and movements, availability of a productive environment) have also been related to maternal education (Defilipo et al., 2012). These two variables were significant predictors of motor development.

Breastfeeding was the second most reliable predictor of cognitive development for the very preterm group and the strongest and only predictor for language and motor development of moderate/late preterm groups. The longer the breastfeeding duration, the higher the cognitive, language, and motor development of those infants. Breastfeeding has been long recognized as a predictor of cognitive, language, and motor development (Månsson et al., 2015), similar to our results. During breastfeeding, mother-infant quality interactions (Silva and Porto, 2016; White-Traut et al., 2018; Rocha et al., 2019) and maternal responsiveness (e.g., staring, touching, talking) to preterm infant behavior are plausible explanations for those results. On the other hand, the lack of mother-preterm infant interactions due to adverse conditions related to prematurity (e.g., hemodynamic instability, respiratory and neurological complications, prolonged hospitalization, mechanical ventilation) restrict the development process.

Our results lead us to acknowledge the specific risk and protective factors associated with neurodevelopment outcomes in the different levels of prematurity, an expected result in this preterm population. As presented in the results, the lower the gestational age, the greater were the associations with biological risk factors. On the contrary, as gestational age increases, environmental factors become more relevant. Extremely infants are usually more affected by severe clinical conditions, requiring several interventions during the NICU stay. Thus, the birth risks and clinical adversity overlap with environmental factors; even if the environment positively affects their neurodevelopment, the established disadvantages are possibly enduring in the neurodevelopment’s harmful effect. Nevertheless, maternal education plays an influential protective role in language and motor development and plays with the infant in motor development, even in this unfavorable condition. As the gestational age increases, we observed that for very preterm, the environment gain intensity in protecting infant cognitive development – maternal education was a protective factor for overall neurodevelopment and play with infant remains a strong positive influence in motor development. Regarding the moderate/late preterm, infants have milder clinical conditions, fewer risk factors were associated with the outcomes, as hypothesized, and protective factors – prolonged breastfeeding positively impact language and motor development.

### Strength and Limitations

The strengths of this study lie in examining neurodevelopmental trajectories in the extremely, very, and moderate/late preterm infants from an LMIC (Low-Moderate Income Countries); longitudinal data for preterm infants from LMIC still feeble. One other strength concerns the fact that we have examined not only risk factors but several environmental factors (environment factors, parental knowledge, and practices); those factors are still under-examined in several countries, from high- to low-income economics, due to its complexity. Therefore, it is the first longitudinal study to show that some features combined of the infants’ early rearing in the home, such as parents’ engagement in play and breastfeeding, were protective factors for the neurodevelopment of preterm infants with several adverse outcomes in LMIC. Another strength is to provide evidence that the infant’s neurodevelopment observed in the first year of life, particularly at 8 months, was highly associated with the infant neurodevelopment at 18 and 24 months. The results suggest the observation of red flags early in life for extremely-, very-, and moderate/late- preterm infants from LMIC.

One weakness concerns the fact that we faced a common difficulty when carrying out longitudinal studies. The missing subjects throughout the longitudinal assessments and consequence uneven distribution of the groups were limitations of the present study. Overall missing data were due to the family’s mobility to other cities to find better job opportunities, parental withdrawal from the follow-up clinic, and mothers joining the workforce. The missing data restrict our capability to examine the environmental factor effect at each specific age (12, 18, and 24 months), and environmental factors could affect children neurodevelopmental differently across this age. Therefore, our recommendation for future studies is to assess environmental factors at specific ages to understand their impact on child life deeply. Also, our results can only be translated to similar LMIC populations with the social strata representation, and our results need further replication.

Another limitation is related to the study design. We conducted a cohort study with three preterm groups; however, the lack of at-term infants’ group restraints our capability to compare the preterm group’s developmental trajectories with at-term infants. Therefore, we were unable to obtain information related to the exact age that preterm infants’ trajectories become different from at term and fall behind.

## Conclusion

The developmental trajectories of the very preterm infants in our study indicated that the delays observed at 2 years old in cognitive, language, and motor development were observable early (at 4– and 8-months), suggesting that the red flags for delays in preterm infants’ neurodevelopment could be detected very early. This study reinforces the importance of early detection and long-term follow-up of preterm infants. The results support the use of BSID-III to assess preterm infants at 4– and 8-month-old in follow-up clinics to prevent undesirable outcomes. At this age, several milestones are observed (e.g., crawling, sitting without support, bimanual coordination, eye-hand coordination, syllables and formation of simple words, object permanence, attachment), and the referral to intervention programs could lessen or prevent the undesirable outcomes at 24-months. Specifically, from our results, interventions to provide parental support to maintain exclusive breastfeeding for the first six months of life and training parents to implement a daily routine of active play with the child, with age-appropriate activities, would benefit infants and their families. Early intervention will increase the likelihood of change in motor trajectories and may inhibit the emergence of possible impairments. Cognitive, language, and motor delays observed in the present study at 24-months will likely contribute to further developmental challenges; therefore, our recommendation is the follow-up of preterm infants until preschool age.

Besides the early detection of delays in this heterogeneous population, it is essential to understand better the determinants of infant exposure to many risk factors and the protective factors supporting preterm infants born at different gestational ages. Consequently, our results support the need to investigate the risk and protective factors in specific preterm groups (i.e., extremely, very, moderate/late) in different periods of life. Although adverse outcomes were strong predictors of neurodevelopment in extremely and very preterm infants, environment variables (formal maternal education and having other children at home) and maternal practices (active play) were protective factors that positively influenced the infants’ neurodevelopment trajectory. A similar trend was observed for moderate/late preterm infants regarding breastfeeding, a single protective factor to overall infant development. Therefore, although the adverse outcomes are risks inherent to the preterm populations, programs focused on maternal training could improve the stimulation and quality of opportunities at home for preterm infants and prevent further risks. Advances in access to information regarding infant development and quality of daily care should focus on public policies in developing countries with high prematurity rates.

## Data Availability Statement

The original contributions presented in the study are included in the article/supplementary material, further inquiries can be directed to the corresponding author/s.

## Ethics Statement

The studies involving human participants were reviewed and approved by Comitê de Ética do Hospital de Clínicas de Porto Alegre – Universidade Federal do Rio Grande do Sul. Written informed consent to participate in this study was provided by the participants’ legal guardian/next of kin.

## Author Contributions

NV, LB, and CP contributed to study design, data acquisition and interpretation, and manuscript preparation. NV, BS, RP, and RS contributed equally to manuscript revision, providing significant oversight on the intellectual content. All authors critically reviewed the manuscript and approved the version submitted for publication.

## Conflict of Interest

The authors declare that the research was conducted in the absence of any commercial or financial relationships that could be construed as a potential conflict of interest.

## Publisher’s Note

All claims expressed in this article are solely those of the authors and do not necessarily represent those of their affiliated organizations, or those of the publisher, the editors and the reviewers. Any product that may be evaluated in this article, or claim that may be made by its manufacturer, is not guaranteed or endorsed by the publisher.
